# Rare Variant Burden and Behavioral Phenotypes in Children with Autism in Slovakia

**DOI:** 10.3390/genes16080893

**Published:** 2025-07-28

**Authors:** Gabriela Repiská, Michal Konečný, Gabriela Krasňanská, Hana Celušáková, Ivan Belica, Barbara Rašková, Mária Kopčíková, Petra Keményová, Daniela Ostatníková, Silvia Lakatošová

**Affiliations:** 1 Institute of Physiology, Academic Center for Autism Research, Faculty of Medicine, Comenius University in Bratislava, 813 72 Bratislava, Slovakia; gabriela.repiska@fmed.uniba.sk (G.R.); daniela.ostatnikova@fmed.uniba.sk (D.O.); 2 Laboratory of Genomic Medicine, GHC GENETICS SK s.r.o., 841 04 Bratislava, Slovakia; konecny@ghc.sk (M.K.);; 3 Institute of Biology and Biotechnology, Department of Biology, Faculty of Natural Sciences, University of St. Cyril and Methodius in Trnava, 917 01 Trnava, Slovakia

**Keywords:** autism spectrum disorder, burden score, behavioral phenotype, rare DNA variants, variants with uncertain significance

## Abstract

Background: Autism spectrum disorder (ASD) is a heterogeneous group of neurodevelopmental disorders characterized by a complex, multifactorial etiology with a strong genetic contribution. Our study aimed to evaluate the link between the burden of rare genetic variants within a specific panel of ASD and intellectual disability-associated genes and phenotypic variability in a cohort of children with autism in Slovakia. Methods: Gene burden scores were calculated based on pathogenic, likely pathogenic, and uncertain significance rare DNA variants identified by whole-exome sequencing. We then assessed the effect of three different scoring methods on the variance across 15 psycho-behavioral parameters describing the phenotypic profiles of 117 ASD probands. Results: The burden score showed a significant multivariate effect on the combination of psycho-behavioral parameters. This score was associated with the social affect of ADOS-2, as well as with the socialization domain, and total adaptive behavior scores from the Vineland Adaptive Behavior Scales-3 (VABS). While a score based solely on count of pathogenic and likely pathogenic variants did not show a multivariate effect, incorporating variants of uncertain significance revealed a multivariate effect on two adaptive behavior parameters: daily living skills and total adaptive behavior score (VABS). Conclusions: Our findings partially explain the variability in phenotypic manifestation in our ASD patient cohort, highlighting the importance of considering the cumulative effect of rare genetic variants, including those of uncertain significance, in shaping the diverse clinical presentation of ASD.

## 1. Introduction

Autism spectrum disorder (ASD) has an increasing trend of prevalence according to the World Health Organization (WHO). It represents one of the most common neurodevelopmental conditions affecting daily functioning, with a prevalence of 1–1.8% [1,2]. ASD presents with considerable variability in clinical manifestations, encompassing a wide range of symptom severity, adaptive behavior, intellectual abilities (IQ), executive functions, language abilities, and other characteristics, which is precisely why it is referred to as a spectrum. Adaptive behavior refers to the set of conceptual, social, and practical skills people acquire and use in their daily lives to function independently within society and provides crucial information regarding the functional abilities of individuals with ASD [3]. Intellectual disability is among the most significant predictors of low adaptive behavior in children with ASD [3]. According to the Centers for Disease Control and Prevention [4], among 8-year-old children with ASD whose cognitive ability data were available, 39.6% demonstrated intellectual disability (IQ ≤ 70). Another 24.2% fell within the borderline range (IQ 71–85), while 36.1% exhibited average or superior intellectual functioning (IQ > 85). Another significant predictor of adaptive behavior are executive functions, a set of higher-level cognitive skills that allow us to control and coordinate our thoughts, actions, and emotions to achieve goals. It is estimated that 41% to 78% of children with ASD experience deficits in the executive functions [5]. In an effort to identify the etiology of ASD abnormalities and their variability, the role of genes is being investigated [6,7].

The precise etiology of the disorder remains incompletely understood, and it is categorized as a multifactorial disorder, stemming from an intricate interplay of genetic, environmental, and epigenetic factors [7]. Epidemiological studies consistently highlight a significant genetic predisposition to its development, as evidenced by notably higher concordance rates in monozygotic twins compared to dizygotic twins and in siblings in general [8,9,10]. Furthermore, twofold greater concordance among full siblings than in half siblings strongly confirm the evidence of the substantial genetic contribution, with heritability estimates in various studies ranging from 50% to 90% [11].

Approximately 4–5% of ASD cases are clinically defined as syndromic autism, where the autism phenotype is part of a known, clinically defined genetic syndrome, such as Fragile X syndrome or Tuberous Sclerosis. In about 20% of ASD cases, genetic testing by whole-exome sequencing and/or microarray analysis successfully identifies a causal molecular etiology, most often involving rare DNA variants in autism candidate genes or autism-associated Copy Number Variants (CNVs) [12]. For the remaining 75% of cases, the genetic cause remains unclear; it is hypothesized to involve the cumulative effect of numerous common DNA variants (SNPs), which are challenging to determine [13]. Analyzing the protein pathways of genes where causal DNA variants have been identified helps to elucidate various biological mechanisms associated with the ASD phenotype [14,15]. These genes are essential during neurodevelopment, play a role as transcriptional factors, or are responsible for chromatin remodeling [16]. The SFARI gene database compiles information on genes and DNA variants relevant to autism etiology, with data derived from findings of causal de novo variants in patients with autism or from association studies [17].

In our study, we focused on rare DNA variants in genes strongly associated with ASD and their relationship with psycho-behavioral parameters. This involves the burden of rare variants in a specific panel of genes associated with autism and intellectual disability and evaluation of their link to the phenotypic variability in the cohort of children with autism in Slovakia.

## 2. Materials and Methods

The Ethics Committee of the Faculty of Medicine of Comenius University and the University Hospital in Bratislava, Slovakia has approved the presented study in accordance with the 1964 Declaration of Helsinki and its subsequent amendments. The parents of all children enrolled in this study were informed of the study design and the written informed consent form was signed by both parents or caregivers of the respective child.

### 2.1. Participants and Sample Collection

A total of 117 blood samples from children diagnosed with ASD were included in this study. The cohort consists of 85 boys and 32 girls, age 5.84 ± 0.3. Children with autism as part of a specific syndrome (Down syndrome, Fragile X syndrome, Cornelia de Lange syndrome, Tuberous Sclerosis, etc.) or children with other severe comorbidities (severe physical disability, suspected development of a psychotic illness, selective mutism, attachment disorders, global developmental delay, etc.) were excluded. Whole venous blood was collected during morning hours after the ADOS-2 diagnostic procedure into EDTA tubes. Characterization of the cohort included ASD diagnostic procedures, IQ assessment, determination of executive dysfunctioning and adaptive behavior. A total of 61 children were completely nonverbal, 21 used several words in simple sentences, and 36 were fully verbal. 

### 2.2. Diagnosis of ASD

At the Academic Research Center for Autism, Comenius University Faculty of Medicine in Bratislava, children suspected of ASD by their pediatricians or psychologists underwent diagnosis using diagnostic tools, including the Autism Diagnostic Observation Schedule 2nd revision (ADOS-2) and Autism Diagnostic Interview-Revised (ADI-R), in line with Diagnostic and Statistical Manual of Mental Disorders 5th edition (DSM-5) by a trained psychologist [18,19]. Within the ADOS-2, children were diagnosed using modules 1, 2, and 3 according to their language deficits and age. Calibrated scores for social affect (SA), restrictive and repetitive behaviors (RRBs), and total calibrated severity scores (CSSs) were used as a measure of symptom severities. These scores are corrected for age and developmental level and range from 0 to 10, with the bigger score referring to a more severe symptomatology. ADI-R results comprise the three domains, including qualitative abnormalities in social interaction (domain A), qualitative abnormalities in social communication (domain B), and restricted, repetitive, and stereotyped patterns of behavior (domain C). To account for the potential influence of age on raw scores, the final score for each domain was converted into a ratio ranging from 0 to 1. This ratio represents the child’s raw score divided by the maximum possible score attainable and is then expressed as a percentage (%). A higher percentage indicates a greater severity of symptoms.

### 2.3. Assessment of Adaptive Behavior

The Vineland Adaptive Behavior Scales-3 (VABS-3) questionnaire was used to measure adaptive behavior, defined as activities necessary for functioning in everyday life, or, in other words, an individual’s ability to meet the standards of social responsibilities and independence [20]. The communication domain measures the individual’s ability to communicate verbally and nonverbally, including expressive, receptive, and written communication. The daily living skills domain includes personal (e.g., dressing and hygiene), domestic (e.g., household chores), and community (e.g., managing money) activities. The socialization domain covers interpersonal relationships, play and leisure activities, and coping skills. In statistical analyses, we use separate scores for each of the three listed domains, as well as a single Adaptive Behavior Composite score, which represents the overall level of adaptive behavior. The VABS-3 standard scores have a mean of 100 and standard deviation of 15. Scores ranging from 85 to 115 are considered to be within the normal range, while scores lower than 70 are considered to be within the impaired range of ability. Thus, higher scores represent better adaptive functioning. VABS-3 also assesses maladaptive behaviors through two distinct scales: internalizing and externalizing maladaptive behaviors. Internalizing maladaptive behaviors encompass difficulties like emotional dysregulation, sleep disturbances, and feeding issues. In contrast, externalizing maladaptive behaviors primarily involve aggressive acts, whether verbal or physical, directed towards individuals or objects. To enable comparisons across different age groups, all raw scores are converted into standard scores. The average score for maladaptive behavior on the VABS-3 is 15, with a standard deviation of 3. A higher score indicates greater severity of maladaptive behaviors.

### 2.4. Assessment of Cognitive Abilities and Executive Functions

According to the age, language, behavior, and ability to cooperate during the assessment, the cognitive abilities were evaluated by a standardized test of intellectual abilities: SON–R 2½-7- for non-verbal children, with a mental age of 2.5 years and up [21], or Woodcock–Johnson International Editions II for verbal children older than 5 years [22]. For evaluation of the effect of genetic factors, the total IQ score was taken from these assessments into analysis. Behavior Rating Inventory of Executive Function—Preschool and Second Version for school children (BRIEF-P, BRIEF-2) was used to assess executive dysfunctions in the probands [23]. The questionnaires measure the potential difficulties in day-to-day executive tasks—like planning, shifting, emotional control, working memory, and inhibition. T-score of BRIEF-P or BRIEF-2 as a measure of executive dysfunction was taken into consideration for evaluation of the genetic effects.

### 2.5. Whole Exome Sequencing and Clinical Interpretation

DNA isolation from 200 µL of whole venous blood was performed using the QIAamp DNA Mini Kit (Qiagen, Hilden, Germany) according to the standard procedure. The whole exome sequencing was performed by DNA library preparation using the Sophia DNA Library Preparation Kit II, Whole Exome Solution v2 (Sophia Genetics, Boston, MA, USA), covering the coding regions of 19,425 genes and the entire mitochondrial genome, and then sequenced on a NextSeq 550 System (Illumina, San Diego, CA, USA). After read mapping and data filtering, detailed variant analysis was performed within the software platform Sophia DDM v4 (Sophia Genetics, Boston, MA, USA).

For the analysis of burden of rare variants, a specific virtual panel of 2045 genes was used, which contained genes from SFARI database [17] with the highest degree of relevance to autism, i.e., scores 1, 2, or S, and genes from the PanelApp [24] panel for autism and intellectual disability were also used, with highest degree of relevance, i.e., green mark.

Detected DNA variants in ASD-associated genes meeting quality criteria were subsequently filtered based on their population frequency (GnomAD > 1%) and within the Sophia DDM user database (>5%), with a focus on rare variants. Identified genomic variants were then classified according to American College of Medical Genetics and Genomics/Association for Medical Pathology (ACMG/AMP) guidelines criteria [25] using the Varsome germline variant classifier, which automatically generates a pathogenicity recommendation based on these guidelines and the vast range of machine-readable genomic data using the Varsome database [26].

The burden score of each patient was calculated by summation of ACMG/AMP rule point system [27] for variants with pathogenic (category 5), likely pathogenic (category 4), and uncertain clinical significance (VUS, category 3, only variants with point system score more than 0) available in the Varsome database (Erasmus Medical Center, Rotterdam, Netherlands). Mode of inheritance was determined using the OMIM database [28].

The second score was based on summation of the total number of variants classified as pathogenic or likely pathogenic (COUNT 5,4), according to the Varsome database.

The third score was based on summation of the total number of variants classified as pathogenic, likely pathogenic, and variants of uncertain significance (only variants with point system score more than 0 were included) (COUNT 5,4,3), according to the Varsome database.

### 2.6. Statistical Analysis

Multivariate linear regression was used to assess the effect of the burden score, COUNT 5,4, and COUNT 5,4,3 on variance across multiple dependent variables describing phenotypic profiles of probands, adjusting for covariates including age and sex. Multiple dependent variables included scores of symptom severities measured by ADOS-2 and ADI-R, adaptive behavior domains, IQ, and level of executive dysfunction measured by the BRIEF T-score. In this analysis, psycho-behavioral parameters including IQ and executive dysfunctions were treated as outcome variables, and the extent to which their variability was influenced by genetic factors was examined. Linearity was assessed via residual plots of the regression model. The significance level was set to *p* < 0.05. If the significant multivariate effect was found, tests of between-subject effects were performed with the application of Bonferroni correction of multiple-comparison testing (*p* < 0.003). A Pearson correlation matrix was used to show the intercorrelation between the three measures of gene burden. Statistical tests were performed in SPSS and Jamovi [29,30].

## 3. Results

A total of 117 ASD patients diagnosed in the aAcademic Research Center for Autism, Comenius University Faculty of Medicine in Bratislava were included in the analysis. Using whole exome sequencing, filtering, and annotation focused on rare DNA variants in the selected virtual gene panel, pathogenic, likely pathogenic, and/or variants of uncertain significance were identified in all analysed samples. Identified variants were classified and annotated using the Varsome database and only variants with a Varsome score of more than 0 (computed as the sum of the points from pathogenic rules, minus the sum of the points from benign rules) were included in subsequent analyses (detailed information of all identified variants is available in the Supplementary Material File S1). Subsequently, we proposed potentially causal variants for ASD in our cohort, which are summarized in Table 1. The potentially causal variants included those variants in relevant genes with pathogenic or likely pathogenic clinical predictions, those with an autosomal dominant mode of inheritance in the heterozygous state, or those with an autosomal recessive mode of inheritance in the homozygous or compound-heterozygous state. Variants in genes with X-linked inheritance were not detected. Variants in genes with an unknown mode of inheritance, however, with potential to explain autism based on SFARI gene data, were also included.

After identification and annotation of rare DNA variants, the burden score, COUNT 5,4 score, and COUNT 5,4,3 score of each patient were calculated. The data summarizing the calculated scores and the spectrum of behavioral parameters obtained by psychological testing are shown in Table 2. The studied cohort exhibited, on average, moderate to high levels of ASD symptoms, borderline intellectual functioning, and below-average adaptive behavior. Executive functions were impaired in most of the probands reaching clinical significance (BRIEF T score above 65).

Three different ways of calculating the genetic burden of rare variants were evaluated for their effect on phenotypic variability using multivariate linear regression. The burden score had a statistically significant multivariate effect on the combination of 15 psycho-behavioral parameters after controlling for age and sex (Pillai’s Trace 10.538, *p* = 0.039) (Table 3).

**Figure 1:**
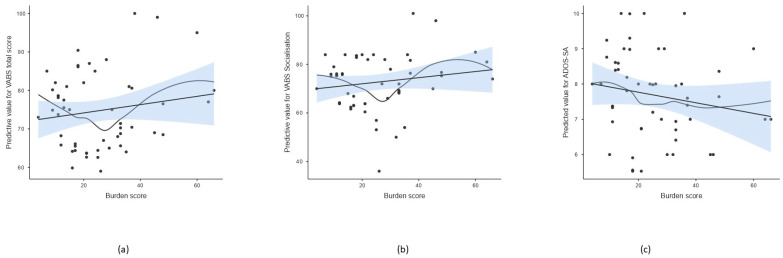
Scatterplot of burden score and predicted value of selected psycho-behavioral parameters after adjusting for age and sex. The line represents the linear regression fit with standard error of the mean (SEM), while the curve indicates a non-parametric smoothed trend line. (**a**) Total score of adaptive behavior measured by VABS, (**b**) socialization scale of VABS, (**c**) social affect of ADOS-2.

Tests of between-subject effects showed that the burden score was associated with the social affect (SA) score and calibrated severity score (CSS) of ADOS-2, daily living skills, socialization, and total adaptive behavior scores of VABS; however, the effect on ADOS-CSS and VABS daily living skills did not remain significant after the multiple comparison correction (Table 4, Figure 1). Linearity was assessed using residual plots. The residuals appeared randomly scattered around zero across fitted values and the burden score variable, indicating no substantial evidence of non-linearity. In addition, a non-parametric smooth line was included in the scatterplot for visualizing potential non-linear trends (Figure 1). The burden score explained the largest proportion of the variability in total adaptive behavior and socialization measured by VABS and social affect measured by ADOS-2 (R^2^ = 0.666, 0.580, 0.577, respectively).

**Figure 2:**
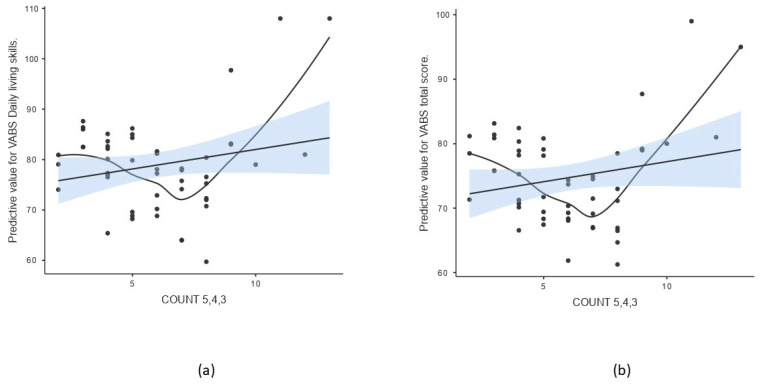
Scatterplot of COUNT 5,4,3 and predicted value of selected psycho-behavioral parameters after adjusting for age and sex. The line represents the linear regression fit with standard error of the mean (SEM), while the curve indicates a non-parametric smoothed trend line (**a**) daily living skills scale of VABS, (**b**) total score of adaptive behavior measured by VABS.

The score based on counting of variants with pathogenic and likely pathogenic predictions (COUNT 5,4) did not show a multivariate effect on the spectrum of behavioral parameters adjusted for sex and age (Pillai’s Trace 1.486, *p* = 0.063) (Table 3). When including the number of variants of uncertain significance (COUNT 5,4,3), a multivariate effect was found (Pillai’s Trace 3.836, *p* = 0.042) (Table 3), with two parameters of adaptive behavior being affected significantly after multiple comparison testing: daily living skills and total adaptive behavior score measured by VABS (Table 5, Figure 2). As described previously, linearity was assessed using residual plots, which showed no violations of the linearity assumption. A non-parametric smooth line was added to the scatterplot to visualize potential non-linear trends (Figure 2).

The analysis of relationships between three scoring system has shown that there is a correlation between the burden score, the count of variants classified as pathogenic or likely pathogenic, and the count that also includes variants of uncertain significance (Table 6).

## 4. Discussion

The rise of high-throughput sequencing completely transformed genetic research, allowing scientists to investigate ASD at an exome/genome-wide level. This technology quickly confirmed that ASD’s origins are multigenic and incredibly diverse, with only a small number of affected individuals sharing the same specific disease-causing genetic variations. At the same time, WES analyses of ASD patients bring the possibility to detect rare variants in genes with various clinical effects and in some cases contribute to the clarification of the genetic cause of ASD by clinical geneticists [31]. However, the majority of the cases still remain without a genetic diagnosis, which may be a result of the current limited methodology to capture causal DNA variants and more probably due the polygenic nature of autism. In our study, we employed the standard DNA diagnostic algorithms in analysis of WES data and we identified potentially causal variants in 24 children (20.5%) (Table 1). A total of 46% of these variants were previously reported in patients with several clinical conditions according to the ClinVar database [32]; only one of them, particularly variant chr11-7994466T > G in EIF3F, was reported in a patient with autism [33]. Our results are in concordance with previous studies, where WES has yielded results ranging between 9 and 30% in individuals with ASD [34]. However, the diagnostic yield is most probably lower, since most of these variants were not confirmed by other methods and the segregation analysis of the variants with the disease in the blood relatives of the probands was not yet performed. In addition, although the genes included in the virtual panel were stringently selected, some are associated with multiple clinical phenotypes, which complicates the interpretation and reduces the clarity of the diagnostic yield.

We further attempted to capture the polygenic nature of autism by calculating the gene burden of rare variants in the cohort. Previous studies have shown that the burden testing for ASD represents a promising approach to discover ASD-associated genes and new associations when strict criteria for variant and gene selection were applied [35,36,37,38]. In our study, a specific virtual panel consisting only of genes with highest degree of relevance to autism and intellectual disability was used for identification of variants involved in rare variant burden. Subsequently, three scores were calculated based on DNA variants categorized according to ACMG criteria. Importantly, two scores—the burden score and COUNT 5,4,3—include variants categorized as pathogenic, likely pathogenic, and variants with uncertain significance (VUS), with a Varsome score more than 0. Crucially, this strategy also considers rare variants of uncertain significance (VUS), as they potentially hold clinical significance, but they are not reported in the medical literature as disease-causing ones or included in human genomic databases. This is crucial, because more severe clinical presentations and associated comorbidities typically correlate with a higher genetic burden. These additional hits involve multiple genetic alterations, such as CNVs or rare inherited variants. While these are often classified as VUS in neurotypical individuals, they are found significantly less frequently in that population [39].

It is known that ASD manifests significant pathophysiological differences across individuals. For better understanding of this phenotypic heterogeneity, it is necessary to understand how ASD-associated mutations affect phenotypic characteristics of the disorder. In this study, we investigated the effect of genetic background, represented by calculated scores on selected psychological and behavioral parameters obtained during the diagnostic process in cohort of 117 ASD patients diagnosed within the Academic Research Center for Autism. Despite the limitation of our study, such as absence of a neurotypical control group, preventing us from evaluating if the overall variant burden in our ASD cohort differs from a baseline population, our results primarily highlight within-cohort relationships between genetic burden and phenotypic variability. Multivariate linear regression analysis showed that the burden score had a statistically significant multivariate effect on the combination of 15 tested psycho-behavioral parameters, especially affecting the social behavioral expressions measured by direct observation of the children during ADOS-2 examination (SA domain) and socialization skills measured by the VABS questionnaire (Table 3 and Table 4, Figure 1). Overall level of adaptive behavior was also affected significantly by the burden score, trending effects for daily living skills and communication domain measured by VABS, and overall symptom severity and restrictive and repetitive behaviors measured by ADOS-2. suggesting possible effects that may reach significance with a higher power, e.g., with a larger sample size (Table 4). When analyzing the effect of count of rare variants with pathogenic or likely pathogenic predictions or variants of uncertain significance (COUNT 5,4 and COUNT 5,4,3), only the effect of COUNT 5,4,3 was able to explain the variability in some psycho-behavioral parameters (Table 3). Thus, our results show that VUS variants should not be omitted in evaluations of total gene burden and their number may be important in influencing the phenotypic manifestations in ASD. These variants may be resolved in upcoming years, revealing their clinical effect and bringing more preciseness in the evaluation of gene burden as well [40,41]. In addition, the psycho-behavioral parameters that were mostly influenced by COUNT 5,4,3 were adaptive behavior domains, namely daily living skills and total level of adaptive behavior measured by VABS. The plots displayed in Figure 2 also show a non-linear pattern after adding a non-parametric smooth curve, similar to the burden score effect. These similarities are obvious, since all the three measures of gene burden are in mutual correlation (Table 6). The relationship between genetic burden and several domains of adaptive behavior shows an interesting pattern, suggesting that a genetic burden near the median (approximately median + 0.5 SD) was associated with the most pronounced deficits in adaptive behavior. The curvature of these lines was driven by a subgroup of fifteen patients with a genetic burden near the median who exhibited the most pronounced deficits in adaptive behavior. The variants observed in these individuals’ affected genes were involved in several shared biological pathways, notably membrane ion transport and neuronal signaling. However, a larger sample size would be necessary to clarify the effect of gene burden on individual psych-behavioral parameters. Previous studies explored the changes in adaptive behavior in ASD and their neurobiological correlates, including genes implicated in neuroanatomical changes observed in ASD children with different levels of adaptive behavior, confirming the effect of genetic factors in modulating adaptive behavior [42]. On the other hand, it is noteworthy that adaptive behavior is also strongly influenced by environmental factors [43]. This twin study, for instance, showed a strong effect of shared environment on the development of social communication and motor skills [43]. According to our results, the burden score had an effect on the variability in two social behavior domains tested by independent methods, ADOS-2 SA and VABS socialization. Previous studies attempted to explain neurobiological correlates of social behavior by employing animal models of ASD [44]. They found that many gene defects are linked to autism-like behavior in mice, especially genes that regulate synaptic function, excitatory/inhibitory balance, and neuronal plasticity. Altered function of these genes results in disbalanced perception, impaired learning and cognition, and altered emotional control, leading to altered social behavior [44]. We found rare DNA variants in many of these genes, with pathogenic, likely pathogenic, or uncertain clinical predictions in our cohort (Supplementary Material File S1).

Our results partially explain the variability in phenotypic manifestation in our ASD cohort. However, this heterogeneity is also likely influenced by numerous common variants, with low individual risks and potentially strong cumulative effects [13]. In addition, the incomplete penetrance of certain genetic variations as well as the mutual interplay between rare DNA variants and the polygenic risk score of common variants and the role of environmental factors makes the search for genotype–phenotype relationships in ASD even more challenging. In addition, other studies indicate that functional mutations are more often found in haploinsufficient genes, confirming the crucial role of dosage effects in the disease mechanisms. The functional characteristics of these affected genes, like the brain expression levels, likely influence the resulting phenotypic consequences of de novo ASD mutations. Stronger functional insults lead, on average, to more severe ASD phenotypes [14]. All these factors, together with complex gene–environment interactions, must be considered, because individuals harboring a genetic predisposition (imparted by common or rare genetic variants) may manifest the disorder upon exposure to specific environmental or epigenetic modifiers that perturb neurodevelopment. Epigenetic studies, particularly epigenome-wide association studies (EWASs) of human brain and accessible surrogate tissues, have emerged as a novel way to understand the complex, multivariate etiologies of developmental disorders like ASD. These studies have identified convergent genes that are epigenetically altered in ASD, many of which overlap with established genetic risk factors. These findings highlight how the genome interacts with the environment, providing a unique perspective on the complex causes of both syndromic and non-syndromic ASD [45].

With advancements in genomic technology, bioinformatics, computational predictions, and the growth of human genomic databases, we anticipate learning significantly more about the genetic underpinnings of ASD. However, larger studies are needed to better understand how genetic factors, particularly burden scores and polygenic risk scores, relate to the detailed psycho-behavioral characteristics of individuals with ASD.

## Figures and Tables

**Table 1:** Summary of potentially autism-associated DNA variants in genes of specific virtual panel. het—heterozygote, hom—homozygote, AD—autosomal dominant, AR—autosomal recessive, *—genes associated with more clinical conditions. **—variants detected in the same patient.

Gene	cDNA	Reference Sequence	Zygosity	dbSNP ID	Pathog. (Varsome)	Inheritance (OMIM)
ACTG1	c.221G > C	NM_001614	het	rs11549185	4	AD
BCAS1	c.1351-1del	NM_003657	het	-	4	unknown
COL4A1	c.904G > A	NM_001845	het	rs144795487	4	AD
DIAPH1	c.3149-2_3149-1del	NM_005219	het	rs1491088674	4	AD/AR
DLGAP3	c.2306dup	NM_001080418	het	-	4	unknown
DNM1L	c.1430_1431dup	NM_012062	het	-	4	AD/AR
EIF3F	c.694T > G	NM_003754	hom	rs141976414	4	AR
FBN1 *	c.6449G > A	NM_000138	het	rs794728250	4	AD
MED13	c.68C > T	NM_005121	het	-	4	AD
MLC1 **	c.577C > T	NM_015166	comp-het	rs555304253	4	AR
MLC1 **	c.581T > G	NM_015166	comp-het	-	3	AR
NAA15	c.1645C > T	NM_057175	het	rs1179904078	5	AD
NKX2-1	c.658G > C	NM_001079668	het	-	4	AD
PCDH15	c.4673_4676del	NM_001142769	het	rs483353074	4	AR/DR
PCDH15	c.4601G > A	NM_001142767	het	-	5	AR/DR
RHOBTB2	c.910del	NM_015178	het	rs1317225352	4	AD
SCN1A	c.5113T > C	NM_001165963	het	-	4	AD
SDHA *	c.1283_1298del	NM_004168	het	-	5	AD/AR
SOD1	c.272A > C	NM_000454	het	rs80265967	5	AR/AD
TCF4	c.1706G > A	NM_001083962	het	-	5	AD
TRIO	c.2149C > T	NM_007118	het	-	4	AD
TTN *	c.33183_33187delinsC	NM_001267550	het	-	4	AD/AR
TTN *	c.11183dup	NM_001267550	het	rs778172350	4	AD/AR
WDFY3	c.1080del	NM_014991	het	-	4	AD

**Table 2:** Biological and psycho-behavioral characteristics of the cohort. ADI-R A—domain A: qualitative abnormalities in social interaction (%), ADI-R B—domain B: qualitative abnormalities in social communication (%), ADI-R C—domain C: restricted, repetitive, and stereotyped patterns of behavior (%). ADOS-2 SA: social affect (calibrated score ranged 0–10), ADOS-2 RRB: restrictive and repetitive behavior (calibrated score ranged 0–10), ADOS-2-CSS: overall calibrated severity score (ranged 0–10), BRIEF: total score of executive dysfunctioning, VABS—adaptive behavior domains.

	Burden Score	COUNT 5,4	COUNT 5,4,3	Age	ADI-R A	ADI-R B	ADI-R C	ADOS-2 SA	ADOS-2 RRB	ADOS-2 CSS
Mean ± SEM	23.5 ± 1.29	1.23 ± 0.11	5.62 ± 0.21	5.84 ± 0.299	0.496 ± 0.02	0.550 ± 0.02	0.355 ± 0.02	7.68 ± 0.14	7.97 ± 0.16	7.93 ± 0.13
95% CI	21.0–26.1	1.2–1.44	5.20–6.30	5.25–6.43	0.46–0.53	0.51–0.59	0.32–0.39	7.39–7.96	7.67–8.28	7.67–8.19
	IQ	BRIEF	VABS communication	VABS daily living skills	VABS socialization	VABS total score	VABS motor skills	VABS maladaptive internalizing	VABS maladaptive externalizing	
Mean ± SEM	87.8 ± 2.74	69.7 ± 1.49	70.9 ± 1.86	76.1 ± 1.18	70.2 ± 1.28	72.2 ± 1.6	81.5 ± 1.45	18.8 ± 0.26	18.1 ± 0.26	
95% CI	82.4–93.3	66.7–72.6	67.2–74.6	73.8–78.4	67.7–72.7	70.1–74.3	78.6–84.4	18.2–19.3	17.6–18.6	

**Table 3:** Results of multivariate linear regression analysis using Pillai’s Trace test. F—F-statistics, Hyp df—Hypothesis degrees of freedom, Error df—Error degrees of freedom, *p*—*p*-value.

Effect	Test	Value	F	Hyp df	Error df	*p*
Burden Score	Pillai’s Trace	10.538	1.217	495	255	0.039
COUNT 5,4	Pillai’s Trace	1.486	1.380	60	140	0.063
COUNT 5,4,3	Pillai’s Trace	3.836	1.249	165	358	0.042

**Table 4:** Test of between-subject effects after multivariate linear regression of burden score and fifteen psycho-behavioral parameters. Level of significance * *p* < 0.01.

Psycho-Behavioral Parameter	F	df	*p*	Adjusted R^2^
ADOS-2-SA	3.194	(33)	0.007 *	0.580
ADOS-2-CSS	2.406	(33)	0.029	0.500
VABS Total score	3.828	(33)	0.002 *	0.666
VABS Socialization	3.158	(33)	0.007 *	0.577
VABS Daily Living Skills	2.343	(33)	0.033	0.503

**Table 5:** Test of between-subject effects after multivariate linear regression of COUNT 5,4,3 and fifteen psycho-behavioral parameters. * *p* < 0.01.

Psycho-Behavioral Parameter	F	df	*p*	Adjusted R^2^
VABS Communication	2.480	(11)	0.018	0.351
VABS Daily living skills	3.878	(11)	0.001 *	0.426
VABS Total score	2.863	(11)	0.008	0.322
VABS Maladaptive behavior externalizing	2.471	(11)	0.019	0.250

**Table 6:** Pearson correlation matrix of the three measures of genetic burden (r-Pearson coefficient, *** *p* < 0.001).

	Burden Score	COUNT 5,4	COUNT 5,4,3
Burden score	-		
COUNT 5,4	r = 0.851 ***	-	
COUNT 5,4,3	r = 0.633 ***	r = 0.339 ***	-

## Data Availability

The original contributions presented in this study are included in the article/Supplementary Material. Further inquiries can be directed to the corresponding author(s).

## Supplementary Materials

The following supporting information can be downloaded at: https://www.mdpi.com/article/10.3390/genes16080893/s1. File S1. List and description of rare DNA variants identified in the cohort of 117 ASD patients.
